# Clinical trials portfolio and regulatory outcomes of ixazomib in multiple myeloma: A systematic review and meta‐analysis

**DOI:** 10.1111/bjh.70757

**Published:** 2026-08-23

**Authors:** Veer Shah, Darshi Shah, Payton Utzman, Karan Shah, Srivatsan Swaminathan, Maria Mainou, Rajshekhar Chakraborty, Aaron Goodman, Samer Al Hadidi, Amar H. Kelkar, Edward R. Scheffer Cliff, Ghulam Rehman Mohyuddin

**Affiliations:** ^1^ Icahn School of Medicine at Mount Sinai New York New York USA; ^2^ Renaissance School of Medicine, Stony Brook University Stony Brook New York USA; ^3^ University of Utah School of Medicine Salt Lake City Utah USA; ^4^ The Johns Hopkins University School of Medicine Baltimore Maryland USA; ^5^ Clinical Research and Evidence‐Based Medicine Unit, Second Medical Department Aristotle University of Thessaloniki Thessaloniki Greece; ^6^ Multiple Myeloma and Amyloidosis Program Columbia University Irving Medical Center New York New York USA; ^7^ MountainView Hospital Las Vegas Nevada USA; ^8^ Harold C. Simmons Comprehensive Cancer Center UT Southwestern Medical Center Dallas Texas USA; ^9^ Dana Farber Cancer Institute Boston Massachusetts USA; ^10^ Harvard Medical School Boston Massachusetts USA; ^11^ Department of Clinical Haematology Peter MacCallum Cancer Centre and Royal Melbourne Hospital Melbourne Victoria Australia; ^12^ Sir Peter MacCallum Department of Oncology The University of Melbourne Melbourne Victoria Australia; ^13^ Program on Regulation, Therapeutics and Law Brigham and Women's Hospital, Harvard Medical School Boston Massachusetts USA; ^14^ Division of Hematology, Huntsman Cancer Institute University of Utah Salt Lake City Utah USA

**Keywords:** drug development, drug regulation, ixazomib, me too drug, multiple myeloma, next in class drug, proteasome inhibitor

To the Editor,

Bortezomib and carfilzomib are proteasome inhibitors that are established mainstays of myeloma treatment. By comparison, the development and uptake of ixazomib, the only orally administered proteasome inhibitor, have been mixed, despite ixazomib receiving US Federal Drug Administration (FDA) approval over a decade ago.

Despite numerous clinical trials including >6600 patients, many questions regarding ixazomib's clinical utility remain unanswered. While its oral administration may offer convenience, ixazomib's relative efficacy compared to bortezomib and carfilzomib is unknown, as it has not been compared with them directly in head‐to‐head randomized controlled trials, and concerns have arisen regarding its tolerability and toxicity.^1^

Phase 1 dose‐escalation studies established a recommended dose and demonstrated preliminary single‐agent activity in relapsed myeloma, providing a rationale for further development.^2^ Unfortunately, multiple subsequent ixazomib trials failed to demonstrate improved clinical outcomes. For example, in the GEM2014MAIN trial, the addition of ixazomib to lenalidomide maintenance did not improve progression‐free survival (PFS).^3^ Although the 2015 approval of ixazomib was based on improved PFS in the TOURMALINE‐MM1 trial, this did not translate to improved overall survival (OS) after a median follow‐up of 85 months.^4^, ^5^ Studies in the maintenance setting (TOURMALINE‐MM3 and TOURMALINE‐MM4) demonstrated concerning trends towards inferior OS, prompting a change to the FDA label advising against ixazomib use in the newly diagnosed or maintenance settings.^6^, ^7^, ^8^, ^9^, ^10^

Given uncertainty regarding the safety and efficacy of ixazomib, we evaluated its entire clinical trial portfolio in a systematic review and meta‐analysis. We reviewed the regulatory approach taken and calculated the revenue generated by ixazomib, drawing lessons to inform the development of and regulatory approaches to next‐in‐class anticancer therapies.

We conducted a systematic review and meta‐analysis following Preferred Reporting Items for Systematic Reviews and Meta‐Analyses (PRISMA) guidelines, registered in PROSPERO (CRD420251080385), using a methodological approach modelled on a prior study of idelalisib in non‐Hodgkin lymphoma.^11^ All prospective clinical trials of ixazomib in myeloma opened, reported or published prior to July 2025 were included, whether as manuscripts, conference abstracts or posted on ClinicalTrials.gov. Retrospective studies were excluded. Trials were identified by independently searching ClinicalTrials.gov and PubMed from inception through July 2025.

For each trial, two researchers extracted trial characteristics, primary end‐point(s) and safety outcomes, and determined whether the primary end‐point was met, whether a PFS or OS benefit was demonstrated, and whether the trial design permitted isolation of ixazomib's specific effect. Results were synthesized descriptively and presented as frequencies and medians with interquartile ranges. Pooled risk ratios (RRs) with 95% confidence intervals were calculated for dichotomous outcomes using a Mantel–Haenszel random‐effects model; heterogeneity was assessed with the I^2^ statistic. All analyses used Review Manager v5.4.1.

For the safety meta‐analysis, we used the four TOURMALINE‐MM randomized placebo‐controlled trials, which permitted attribution of adverse events (AEs) to ixazomib. Key adverse events such as nausea, vomiting, rash, peripheral neuropathy, discontinuation due to AEs, grade ≥3 AEs, fatal AEs and serious AEs were pooled across trials using a random‐effects model. Risk of bias in all randomized control trials (RCTs) with reported results was assessed using the Cochrane risk‐of‐bias tool.

Global ixazomib revenue was estimated from Takeda's Form 20‐F SEC filings (2016–2024), converted from Japanese Yen to inflation‐adjusted 2025 United States dollars. Medicare Part D spending was obtained from publicly available Centers for Medicare and Medicaid Services (CMS) data (2019–2023).

We identified 202 studies evaluating ixazomib. After excluding retrospective studies and removing duplicates, 87 unique prospective clinical trials remained, enrolling a total of 6689 patients (4670 patients in randomized trials and 2019 in non‐randomized trials). Figure 1 highlights the clinical development of ixazomib from its first‐in‐human trials to recent RCTs.

**FIGURE 1:**
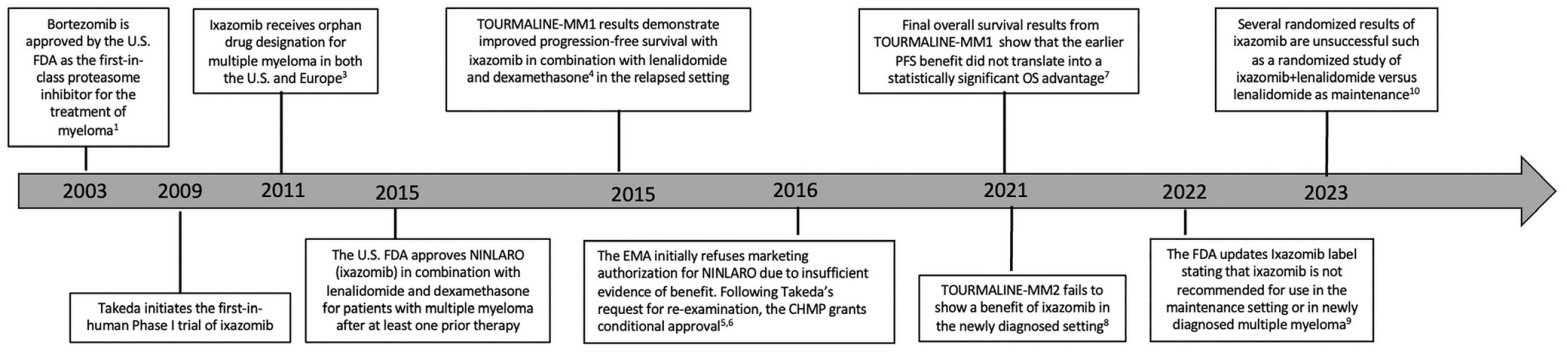
Timeline of ixazomib development and regulatory history.

Of the 87 trials, 56 (64%) had results publicly available, while 31 (36%) had no publicly reported results. 28 trials (32%) were randomized, of which 18 (64%) had fully published results. The median sample size among single‐arm trials was 35 patients (Interquartile range [IQR] 42.5), compared to 127 patients (IQR 262) in RCTs.

Table 1 lists characteristics of included studies, and Table S1 summarizes all RCTs included in our analysis, their characteristics and results.

**TABLE 1:** Characteristics of ixazomib clinical trials.

Category	Subcategory	Count, *n* (%)
Publication status	Complete results published	56 (64%)
	Incomplete/preliminary results	31 (36%)
Study design	RCT	28 (32%)
	Number of patients in RCT	4670 (70%)
	Non‐randomized trials	59 (68%)
	Number of patients in non‐RCT	2019 (30%)
	RCT publication rate	18/28 (64%)
Trial phase	Phase 1	12 (14%)
	Phase 1/2	10 (11%)
	Phase 2	47 (54%)
	Phase 3	9 (10%)
	Phase 4	6 (7%)
	Unknown phase	3 (3%)
	Early phase (1, 1/2, 2)	69 (79%)
Patient population	Newly diagnosed	18 (21%)
	Relapsed/refractory	50 (57%)
	Maintenance therapy	12 (14%)
	Newly diagnosed + maintenance	6 (7%)
	All populations	1 (1%)
Sponsorship	Industry‐sponsored	28 (32%)
	Investigator/academic‐initiated	47 (54%)
	Mixed sponsorship	12 (14%)
Geographic distribution	United States only	43 (51%)
	International only	32 (38%)
	U.S. + International	9 (11%)

Abbreviation: RCT, randomized control trial.

A meta‐analysis of 2778 patients included in the four TOURMALINE RCTs (Table S2) demonstrated a significantly increased risk of AEs leading to treatment discontinuation with ixazomib (pooled RR 1.32, 95% CI 1.12–1.57, *p* < 0.001) and increased grade ≥3 AEs (RR 1.27, 95% CI 1.05–1.53). No significant difference was seen in fatal AEs (RR 0.97, 95% CI 0.67–1.42) or serious AEs (RR 1.11, 95% CI 0.95–1.29). Specific AEs that were significantly increased with ixazomib included diarrhoea (RR 1.37), nausea (RR 1.91), vomiting (RR 2.64), peripheral neuropathy (RR 1.30) and rash (RR 1.71). A meta‐analysis of the four TOURMALINE trials showed no significant OS difference (pooled HR 1.00, 95% CI 0.89–1.12; *p* = 0.99) (Figures S1 and S2).

The Appendix SII lists AEs in detail across all four TOURMALINE RCTs. Table S3 highlights all severe adverse events (SAEs) consistently reported across the four TOURMALINE RCTs, while Table S4 lists ‘Other’ AEs (non‐serious) consistently reported across the 4 RCTs. In addition, Table S5 lists all‐cause mortality, total adverse events (TAEs) and all SAEs across all 4 RCTs.

Of 28 RCTs, 9 (32%) included ixazomib in both arms, precluding robust evaluation of its efficacy or safety. Among 59 non‐randomized trials, ixazomib's effect could not be isolated in 52 (88%). No trial directly compared ixazomib to another proteasome inhibitor and no trial used OS as the primary end‐point. No trial where ixazomib's effect could be isolated demonstrated efficacy of ixazomib in patients with bortezomib‐ or carfilzomib‐refractory disease. Among 18 RCTs with published results, PFS was the most common primary end‐point (13 studies, 72%), of which only 5 (38%) demonstrated improved PFS. Overall, only 6 of 18 RCTs with results posted (33%) met their primary end‐point, compared to 25 of 38 non‐randomized trials (66%) with results.

From 2016 to 2024, ixazomib generated approximately $5.6 billion in global revenue, with annual revenue peaking at $922 million in 2020 before declining to $609 million by 2024. Medicare Part D spending totalled approximately $1.7 billion over 2019–2023 (Table S6) and did not show a parallel decline, potentially suggesting continued widespread use in the United States despite the highly uncertain evidence base.

After 6689 patients were enrolled in 87 clinical trials of ixazomib over >10 years, its utility in the clinic remains unclear. Whether it is as safe or effective as its fellow proteasome inhibitors is unknown, and it seems—based on small datasets—to have limited clinical activity in patients with proteasome inhibitor‐refractory disease.^12^ Its use as maintenance therapy offers limited benefit but significant toxicity, as highlighted by our meta‐analysis of 2754 patients across four TOURMALINE RCTs. Despite its toxicity and concerns regarding inferior OS with ixazomib maintenance, ixazomib continues to be listed as a treatment option for newly diagnosed myeloma and as maintenance therapy in the National Comprehensive Cancer Network guidelines.^10^, ^13^, ^14^

Ixazomib's regulatory journey and development highlight the need for close regulation of the development of next‐in‐class drugs.^15^ Next‐in‐class drugs can be useful if they offer an advance in efficacy, improved safety (or convenience) with similar efficacy, or target disease resistant to an earlier‐in‐class drug. In order to ascertain whether a next‐in‐class drug offers such benefits it must either be tested in randomized trials against the earlier‐in‐class competitor or demonstrate efficacy in patients with disease refractory to the earlier‐in‐class drug. Neither has been demonstrated for ixazomib. This leaves uncertainty whether it is as or less effective and safe than bortezomib and carfilzomib. Stronger regulatory approaches are needed to ensure that clinical trials are designed to answer these critical questions, including head‐to‐head studies. This problem is not unique to ixazomib; the lack of head‐to‐head trials between immunomodulatory drugs leaves similar unanswered questions.

The 59 non‐randomized trials of ixazomib serve as a reminder of the potentially limited utility of single‐arm trials. While single‐arm trials are useful in early drug development to investigate safety, dosing and safety of drug combinations, their use should decrease after initial phase I and II trials.

Rapid clinical adoption of ixazomib as an oral alternative to bortezomib, ahead of robust comparative evidence, underscores that clinicians, not only regulators and manufacturers, bear responsibility for scrutinizing a next‐in‐class drug's evidence base before prescribing it widely.

Limitations of our study include lack of access to patient level data to permit a granular analysis of toxicity. Despite a thorough search strategy, it is possible that some ixazomib studies were not included.

After 6689 patients enrolled across 87 trials, ixazomib has not demonstrated OS benefit in an RCT and its clinical utility remains uncertain. Although millions of dollars have been spent on ixazomib trials, including government‐funded trials, and billions of dollars more on its routine use, its evidence base is insufficient to answer key clinical questions. Its development illustrates the need for robust, proactive regulatory oversight of next‐in‐class drug development to ensure that every clinical trial is generating scientific knowledge that advances the field.

## AUTHOR CONTRIBUTIONS

**Veer Shah:** Data curation; investigation; formal analysis; visualization; writing – original draft; writing – review and editing; validation; software; project administration. **Maria Mainou:** Methodology; formal analysis; validation; visualization; writing – review and editing. **Darshi Shah:** Investigation; formal analysis; data curation; writing – original draft; writing – review and editing; project administration; visualization. **Samer Al Hadidi:** Investigation; validation; writing – review and editing. **Edward R. Scheffer Cliff:** Investigation; validation; writing – review and editing. **Ghulam Rehman Mohyuddin:** Conceptualization; methodology; project administration; writing – review and editing; investigation; supervision; validation; writing – original draft. **Karan Shah:** Writing – original draft; data curation; formal analysis; visualization; writing – review and editing. **Aaron Goodman:** Investigation; validation; writing – review and editing. **Amar H. Kelkar:** Investigation; validation; writing – review and editing. **Rajshekhar Chakraborty:** Writing – review and editing; validation; investigation. **Payton Utzman:** Data curation; formal analysis; writing – original draft; writing – review and editing. **Srivatsan Swaminathan:** Writing – original draft; formal analysis; writing – review and editing.

## FUNDING INFORMATION

This study received no dedicated funding.

## CONFLICT OF INTEREST STATEMENT

Ghulam R. Mohyuddin reports receiving honoraria from writing for MashupMD and Medscape and research funding to his site from Janssen and Sanofi. Rajshekhar Chakraborty reports consulting/advisory roles with Alexion (AstraZeneca), Janssen, Sanofi Pasteur, Pfizer and Adaptive Biotech; travel support from Prothena; and research funding from AbbVie, Genentech, Janssen and StandUp2Cancer. Aaron Goodman reports receiving honoraria from EUSA Pharma and Seattle Genetics. Samer Al Hadidi reports consulting/advisory roles with Janssen, Sanofi and Pfizer, as well as research funding from Alexion, Pfizer and Janssen. Edward R. Scheffer Cliff reports institutional research funding from Arnold Ventures. All other authors report no conflicts of interest.

## Supporting information


**Table S1.** Summary of clinical trials included in the analysis, with study design, disease category, Phase, treatment regimens and primary outcomes.

**Table S2.** TOURMALINE‐MM series of large randomized clinical trials.
**Table S3:** Serious adverse events that are consistently reported across all four TOURMALINE Clinical Trials. Source: clinicaltrials.gov.
**Table S4:** ‘Other’ (non‐serious) adverse events that are consistently reported across All FOUR TOURMALINE clinical trials. Source: clinicaltrials.gov.
**Table S5:** Overall safety data summary. Source: clinicaltrials.gov.
**Table S6:** Ixazomib revenue from 2016 to 2024.

**Figure S1:** Forest plot of common non‐haematological all grade toxicities of ixazomib versus placebo.

**Figure S2:** Forest plots of serious adverse events of ixazomib versus placebo.

## Data Availability

Data are publicly available. Aggregated data may be shared upon reasonable request to the corresponding author.
