# Supplementary material for: Clinical trials portfolio and regulatory outcomes of ixazomib in multiple myeloma: A systematic review and meta‐analysis
Source: Br J Haematol. 2026 Aug 23;209(4):1882–5. doi: 10.1111/bjh.70757 (PMC13674204; doi:10.1111/bjh.70757)
Supplement: Supplementary file 1 [file BJH-209-1882-s001.docx]

**Supplementary Table 1: Summary of Clinical Trials Included in the Analysis, with Study Design, Disease Category, Phase, Treatment Regimens, and Primary Outcomes**

| **Title** | **Disease Category** | **Randomized?** | **Trial Phase** | **Experimental Arm Regimen** | **Control Arm Regimen** | **Sample Size Experimental Arm** | **Sample Size Control Arm** | **Primary Endpoint(s)** | **Primary endpoint met?** |
| --- | --- | --- | --- | --- | --- | --- | --- | --- | --- |
| Comparison between ixazomib+cyclophosphamide+dexamethasone regimen and ixazomib+dexamethasone regimen for elderly and frail patients having newly diagnosed multiple myeloma. (Li *et al.* 2023)^1^ | NDMM | Y | N/A | ixazomib+cyclophosphamide+ dexamethasone | No placebo. Compared to ixazomib+dexamethasone | 19 | 17 | Response Rate | N |
| Final Overall Survival Analysis of the TOURMALINE-MM1 Phase III Trial of Ixazomib, Lenalidomide, and Dexamethasone in Patients With Relapsed or Refractory Multiple Myeloma. (Richardson et al., 2021)^2^ | R/R | Y | Ph3 | ixazomib+  lenalidomide +  dexamethasone | Placebo + lenalidomide + dexamethasone | 361 | 359 | Progression Free Survival | Y |
| Ixazomib or Lenalidomide combined with cyclophosphamide and dexamethasone in the treatment of elderly transplant-ineligible newly diagnosed multiple myeloma. (Wang et al., 2025)^3^ | NMDD | Y | Ph4 | Ixazomib/cyclophosphamide/dexamethasone | lenalidomide/cyclophosphamide/dexamethasone | 31 | 32 | Safety, Response Rate | N |
| Ixazomib as consolidation and maintenance versus observation in patients with relapsed multiple myeloma eligible for salvage autologous stem-cell transplantation (Myeloma XII [ACCoRD]): interim analysis of a multicentre, open-label, randomised, phase 3 trial. (Cook at al., 2024)^4^ | R/R | Y | Ph3 | Ixazomib, Thalidomide, Dexamethasone (2 cycles consolidation) → Ixazomib maintenance (until progression) | Observation (no active therapy) | 103 | 103 | Progression Free Survival | Y |
| Ixazomib with cyclophosphamide and dexamethasone in relapsed or refractory myeloma: MUKeight phase II randomised controlled trial results. (Auner, 2022)^5^ | R/R | Y | Ph2 | Ixazomib + Cyclophosphamide + Dexamethasone | Cyclophosphamide + Dexamethasone | 58 | 54 | Progression Free Survival | N |
| Phase 1 study of twice-weekly ixazomib, an oral proteasome inhibitor, in relapsed/refractory multiple myeloma patients. (Richardson et al,, 2014)^6^ | R/R | N | Ph1 | ixazomib | NA | 60 | NA | Safety | Y |
| Phase 1 study of weekly dosing with the investigational oral proteasome inhibitor ixazomib in relapsed/refractory multiple myeloma. (Kumar et al., 2014)^7^ | R/R | N | Ph1 | Ixazomib | NA | 60 | NA | Safety | Y |
| Ixazomib, lenalidomide, and dexamethasone in patients with newly diagnosed multiple myeloma: long-term follow-up including ixazomib maintenance. (Kumar et al., 2019)^8^ | NMDD | N | Ph2 | Ixazomib + Lenalidomide + Dexamethasone | NA | 65 | NA | Response Rate, Safety | Y |
| A phase I/II dose-escalation study investigating all-oral ixazomib-melphalan-prednisone induction followed by single-agent ixazomib maintenance in transplant-ineligible newly diagnosed multiple myeloma. (San-Miguel et al., 2018)^9^ | NMDD | N | Ph1/2 | Ixazomib + Melphalan + Prednisone (induction), followed by single-agent Ixazomib (maintenance at Recommended Phase II dose) | NA | 61 | NA | Response Rate | Y |
| Twice-weekly ixazomib in combination with lenalidomide-dexamethasone in patients with newly diagnosed multiple myeloma. (Richardson et al., 2018)^10^ | NMDD | N | Ph2 | ixazomib, lenalidomide, and dexamethasone | NA | 64 | NA | Safety, Response Rate | Y |
| Randomized phase 2 trial of ixazomib and dexamethasone in relapsed multiple myeloma not refractory to bortezomib (Kumar et al, 2016).^11^ | R/R | Y | Ph2 | Ixazomib 5.5 mg weekly + Dexamethasone same as above | Ixazomib 4 mg weekly + Dexamethasone | 35 | 35 | Response Rate | Y |
| Pharmacokinetics and safety of ixazomib plus lenalidomide-dexamethasone in Asian patients with relapsed/refractory myeloma: a phase 1 study. (Gupta et al., 2015)^12^ | R/R | N | Ph1 | ixazomib, lenalidomide, dexamethasone | NA | 43 | NA | Other(s), Safety | Y |
| Phase II Study of the Combination of MLN 9708 With Lenalidomide as Maintenance Therapy Post Autologous Stem Cell Transplant in Patients With Multiple Myeloma (Shah et al., 2013)^13^ | Maintainence | N | Ph2 | (Ixazomib) and Lenalidomide | NA | 64 | NA | Progression Free Survival | Y |
| A pharmacokinetics and safety phase 1/1b study of oral ixazomib in patients with multiple myeloma and severe renal impairment or end-stage renal disease requiring haemodialysis. (Gupta et al., 2016)^14^ | R/R | N | Ph1 | Ixazomib ± Dexamethasone | NA | 41 | NA | Other(s) | Y |
| Oral ixazomib, lenalidomide, and dexamethasone for transplant-ineligible patients with newly diagnosed multiple myeloma (Facon et al., 2021)^15^ | NMDD | Y | Ph3 | Ixazomib + Lenalidomide + Dexamethasone | Placebo + Lenalidomide + Dexamethasone | 351 | 354 | Progression Free Survival | N |
| Phase 1/2 trial of ixazomib, cyclophosphamide and dexamethasone in patients with previously untreated symptomatic multiple myeloma (Kumar et al., 2018).^16^ | NMDD | N | Ph2 | Ixazomib + Cyclophosphamide + Dexamethasone | NA | 48 | NA | Safety, Response Rate | Y |
| All-oral triplet combination of ixazomib, lenalidomide, and dexamethasone in newly diagnosed transplant-eligible multiple myeloma patients: final results of the phase II IFM 2013-06 study (Touzeau et al., 2022).^17^ | NMDD | N | Ph2 | Ixazomib + Lenalidomide + Dexamethasone | NA | 42 | NA | Response Rate | Y |
| Alliance A061202: ixazomib, pomalidomide, and dexamethasone for patients with lenalidomide-refractory MM in first relapse (Voorhees at al., 2024).^18^ | R/R | Y | Ph2 | Ixazomib + Pomalidomide + Dexamethasone | Pomalidomide + Dexamethasone | 38 | 39 | Progression Free Survival | Y |
| Phase 2 study of all-oral ixazomib, cyclophosphamide and low-dose dexamethasone for relapsed/refractory multiple myeloma. (Kumar et al., 2019)^19^ | R/R | Y | Ph2 | Ixazomib + Cyclophosphamide + Dexamethasone Randomized to different dosing | NA | 78 | NA | Response Rate | N |
| A Phase I Study of Ixazomib in Combination with Panobinostat and Dexamethasone in Patients with Relapsed or Refractory Multiple Myeloma (Reu et al., 2015)^20^ | R/R | N | 5 = Ph 1/2 | Panobinostat, Dexamethasone, Ixazomib | NA | 16 | NA | Safety | Y |
| Phase I/II of the oral regimen ixazomib, pomalidomide, and dexamethasone in relapsed/refractory multiple myeloma (Krishnan et al., 2018)^21^ | R/R | N | Ph1/2 | ixazomib +dexamethasone and pomalidomide | NA | 32 | NA | Safety, Response Rate | Y |
| Open-Label Study to Determine the Feasibility of Ixazomib as Maintenance after Allogeneic Stem Cell Transplant for Multiple Myeloma (Bachier et al., 2018)^22^ | Maintainence | N | Ph2 | ixazomib | NA | 9 | NA | Safety | Y |
| Deepening responses associated with improved progression-free survival with ixazomib versus placebo as posttransplant maintenance in multiple myeloma. (Goldschmidt et al., 2020)^23^ | NMDD, Maintainence | Y | Ph3 | Ixazomib citrate | Placebo | 395 | 261 | Progression Free Survival | Y |
| A phase 1/2 study of ixazomib in place of bortezomib or carfilzomib in a subsequent line of therapy for patients with multiple myeloma refractory to their last bortezomib or carfilzomib combination regimen (Daniely et al., 2022).^24^ | R/R | N | Ph1/2 | Experimental Arm Drug Regimen/Combination: Various combinations of ixazomib replacing bortezomib or carfilzomib in previously used multi-agent regimens. These included combinations with: dexamethasone lenalidomide pomalidomide cyclophosphamide melphalan prednisone ascorbic acid pegylated liposomal doxorubicin | NA | 46 | NA | Response Rate, Safety | N |
| Ixazomib, lenalidomide and dexamethasone consolidation with randomized ixazomib or lenalidomide maintenance after autologous transplant in newly diagnosed multiple myeloma. (Slade et al., 2022)^25^ | NMDD | Y | Ph2 | Ixazomib, lenalidomide, and dexamethasone (IRd) for consolidation; followed by randomized maintenance with either ixazomib or lenalidomide. | lenalidomide | 94 | 95 | Progression Free Survival | N |
| Ixazomib as Postinduction Maintenance for Patients With Newly Diagnosed Multiple Myeloma Not Undergoing Autologous Stem Cell Transplantation: The Phase III TOURMALINE-MM4 Trial. (Dimopoulos et al., 2020)^26^ | NMDD, maintainence | Y | Ph3 | Ixazomib | Placebo | 425 | 281 | Progression Free Survival | Y |
| Lenalidomide and dexamethasone maintenance with or without ixazomib, tailored by residual disease status in myeloma. (Rosinol et al., 2023)^27^ | maintenance | Y | Ph3 | Ixazomib + Lenalidomide + Dexamethasone (IRD) | Lenalidomide + Dexamethasone (RD) | 171 | 161 | Progression Free Survival | N |
| Ixazomib-Thalidomide-Dexamethasone for induction therapy followed by Ixazomib maintenance treatment in patients with relapsed/refractory multiple myeloma. (Ludwig et al., 2019)^28^ | R/R | N | Ph2 | Ixazomib, Thalidomide, Dexamethasone | NA | 90 | NA | Progression Free Survival | Cannot be ascertained |
| Ixazomib, Oral Metronomic Cyclophosphamide, and Dexamethasone for First-Line Treatment of Multiple Myeloma: A Phase II Brown University Oncology Group Study. (Pelcovits et al, 2023)^29^ | NMDD | N | Ph2 | Ixazomib + Metronomic Cyclophosphamide + Dexamethasone | NA | 12 | NA | Response Rate | Cannot be ascertained |
| A Multicenter Phase II, Double-Blind, Placebo-Controlled Trial of Maintenance Ixazomib After Allogeneic Transplantation for High-Risk Multiple Myeloma: Results of the Blood and Marrow Transplant Clinical Trials Network 1302 Trial. (Bashir et al., 2023)^30^ | maintenance | Y | Ph2 | Ixazomib maintenance | Placebo | 21 | 22 | Progression Free Survival | N |
| Phase I/II trial of bendamustine, ixazomib, and dexamethasone in relapsed/refractory multiple myeloma. (Dhakal et al., 2019)^31^ | R/R | N | Ph1/2 | Bendamustine + Ixazomib + Dexamethasone | NA | 28 | NA | Response Rate, Safety | Y |
| Phase II Prospective Evaluation of Bone Remodeling During Ixazomib Treatment Relapsed/Refractory Multiple Myeloma Patients (NCT02499081) | R/R | N | Ph2 | Ixazomib | NA | 20 | NA | Other(s) | Y |
| Phase 2 Trial of Pomalidomide, Ixazomib and Dexamethasone in Patients With Multiple Myeloma With Extramedullary Disease or Plasma Cell Leukemia (Buadi et al., 2020)^32^ | R/R | N | Ph2 | ixazomib citrate, pomalidomide, dexamethasone | NA | 17 | NA | Response Rate | Cannot be ascertained |
| Ixazomib-based induction regimens plus ixazomib maintenance in transplant-ineligible, newly diagnosed multiple myeloma: the phase II, multi-arm, randomized UNITO-EMN10 trial. (Mina et al., 2021)^33^ | NMDD | Y | Ph2 | ICd = Ixazomib + Cyclophosphamide + Dexamethasone  ITd = Ixazomib + Thalidomide + Dexamethasone  IBd = Ixazomib + Bendamustine + Dexamethasone | Id = Ixazomib + Dexamethasone | 129 | 42 | Progression Free Survival | N |
| Alternating the Administration of Ixazomib and Lenalidomide as Maintenance Therapy After Autologous Transplant for Treating Multiple Myeloma (Holmberg et al., 2022)^34^ | maintenance | N | Ph2 | Ixazomib + Lenalidomide | NA | 30 | NA | Safety | Y |
| Phase 1 / 2 Trial of Idasanutlin in Combination With Ixazomib and Dexamethasone in Patients With 17p Deleted, Relapsed Multiple Myeloma (NCT02633059) | R/R | N | Ph1/2 | Dexamethasone + Idasanutlin + Ixazomib | NA | 33 | NA | Safety | Y |
| Phase II Trial of Ixazomib and Dexamethasone Versus Ixazomib, Dexamethasone and Lenalidomide, Randomized With NFKB2 Rearrangement. (Proteasome Inhibitor NFKB2 Rearrangement Driven Trial, PINR) (Bernal-Mizrachi et al., 2018)^35^ | R/R | Y | Ph2 | Experimental Arm A: Ixazomib + Dexamethasone; Experiemental for NFKB2-mutated patients; Arm B: Ixazomib + Dexamethasone + Lenalidomide for NFKB2-mutated patients | Active comparator arm: Ixazomib + Dexamethasone (NFKB2-unmutated patients) | 43 | 27 | Response Rate | N |
| A phase 2, open-label, multicenter study of ixazomib plus lenalidomide and dexamethasone in adult Japanese patients with relapsed and/or refractory multiple myeloma. (Iida et al., 2022)^36^ | R/R | N | Ph2 | ixazomib+ lenalidomide + dexamethasone | NA | 34 | NA | Response Rate | Y |
| Phase 2 Trial of Ixazomib, Lenalidomide, Dexamethasone, and Daratumumab in Patients With Newly Diagnosed Multiple Myeloma (Kumar et al., 2018)^37^ | NMDD | N | Ph2 | Daratumumab + Dexamethasone + Ixazomib + Lenalidomide | NA | 40 | NA | Response Rate | N |
| Phase 2 Trial of Induction With Ixazomib, Pomalidomide, Dexamethasone Prior to Salvage Autologous Stem Cell Transplant Followed by Consolidation With Ixazomib, Pomalidomide, and Dexamethasone and Ixazomib Maintenance in Multiple Myeloma (NCT03202628) | R/R | N | Ph2 | Dexamethasone + Ixazomib + Pomalidomide | NA | 8 | NA | Progression Free Survival | Cannot be ascertained |
| Efficacy and Safety of Ixazomib Plus Lenalidomide and Dexamethasone Following Injectable PI-Based Therapy in Relapsed/Refractory Multiple Myeloma (Abe et al., 2023)^38^ | R/R | N | Ph4 | [VRd]; Bortezomib + Lenalidomide + Dexamethasone followed by Ixazomib   OR   [KRd]; Carfilzomib + Lenalidomide + Dexamethasone Therapy followed by Ixazomib | NA | 45 | NA | Other(s) | N |
| Ixazomib plus daratumumab and dexamethasone: Final analysis of a phase 2 study among patients with relapsed/refractory multiple myeloma. (Delimpasi et al., 2024)^39^ | R/R | N | Ph2 | Ixazomib + Daratumumab + Dexamethasone | NA | 61 | 0 | Response Rate | Y |
| Abatacept, Ixazomib Citrate, and Dexamethasone in Treating Patients With Multiple Myeloma Resistant to Chemotherapy (NCT03457142) | R/R | N | Ph2 | (abatacept, ixazomib citrate, dexamethasone | NA | 15 | NA | Response Rate | Cannot be ascertained |
| Phase 2 Trial of Pembrolizumab, Ixazomib, and Dexamethasone for Relapsed Multiple Myeloma (NCT03506360) | R/R | N | Ph2 | ixazomib citrate, pembrolizumab, dexamethasone | NA | 13 | NA | Response Rate | Cannot be ascertained |
| Ixazomib Maintenance Therapy in Chinese Patients with Newly Diagnosed Multiple Myeloma Not Treated with Stem Cell Transplantation: A Single-Arm, Open-Label Study (Fu et al., 2023)^40^ | NMDD | N | Ph3 | Ixazomib | placebo | 35 | 4 | Safety | Y |
| MLN9708 (Ixazomib) and MLN4924 (Pevonedistat) in Relapsed/Refractory Multiple Myeloma Patients: A Phase 1b Trial (NCT03770260) | R/R | N | Ph1 | ixazomib citrate, pevonedistat | NA | 8 | NA | Response Rate, Safety | Y |
| Iberdomide, ixazomib and dexamethasone in elderly patients with multiple myeloma at first relapse .(Touzeau et al., 2025)^41^ | R/R | N | Ph2 | Ixazomib, Iberdomide, Dexamethasone | NA | 75 | NA | Response Rate | N |
| Ixazomib, Daratumumab, and Low-Dose Dexamethasone in Frail Patients With Newly Diagnosed Multiple Myeloma: The Hovon 143 Study. (Stege et al, 2021)^42^ | NMDD | N | Ph2 | ixazomib-daratumumab-low-dose-dexamethasone | NA | 65 | NA | Response Rate | Y |
| Ixazomib-Thalidomide-low dose dexamethasone induction followed by maintenance therapy with ixazomib or placebo in newly diagnosed multiple myeloma patients not eligible for autologous stem cell transplantation; results from the randomized phase II HOVON-126/NMSG 21.13 trial (Zweegman et al., 2020)^43^ | maintenance | Y | Ph2 | Ixazomib maintenance after induction with ITd (ixazomib, thalidomide, dexamethasone) | Placebo maintenance after induction with ITd (ixazomib, thalidomide, dexamethasone) | 39 | 39 | Progression Free Survival | N |
| A phase II trial of continuous ixazomib, thalidomide and dexamethasone for relapsed and/or refractory multiple myeloma: the Australasian Myeloma Research Consortium (AMaRC) 16-02 trial. (Bergin et al., 2021)^44^ | R/R | N | Ph2 | Ixazomib, Thalidomide, Dexamethasone | NA | 39 | NA | Response Rate | N |
| Phase I Study of Selinexor, Ixazomib, and Low-dose Dexamethasone in Patients With Relapsed or Refractory Multiple Myeloma. (Salcedo et al., 2020)^45^ | R/R | N | Ph1 | selinexor + ixazomib + dexamethasone | NA | 18 | NA | Safety | Y |
| Multicenter Open Label Phase 2 Single Arm Study of Ixazomib, Pomalidomide and Dexamethasone in Relapsed or Refractory Multiple Myeloma Characterized with Genomic Abnormalities of Adverse Prognostic | R/R | N | 2 = Ph2 | Ixazomib/Pomalidomide/Dexamethasone | NA | 26 | NA | Other(s) | Y |
| Oral ixazomib-dexamethasone vs oral pomalidomide-dexamethasone for lenalidomide-refractory, proteasome inhibitor-exposed multiple myeloma: a randomized Phase 2 trial. (Dimopoulos et al., 2022)^46^ | R/R | Y | Ph2 | Ixazomib + Dexamethasone | Pomalidomide + Dexamethasone | 73 | 49 | Progression Free Survival | N |
| Efficacy and safety of daratumumab with ixazomib and dexamethasone in lenalidomide-exposed patients after one prior line of therapy: Final results of the phase 2 study DARIA. (Terpos et al., 2024)^47^ | R/R | N | Ph2 | Daratumumab + Ixazomib + Dexamethasone | NA | 50 | 0 | Response Rate | Y |
| Increased Bone Volume by Ixazomib in Multiple Myeloma: 3-Month Results from an Open Label Phase 2 Study. (Diaz-delCastillo et al., 2023)^48^ | maintenance | N | Ph2 | Ixazomib | NA | 30 | NA | Other(s) | Cannot be ascertained |
| A Prospective Phase 2 Study to Assess the Minimal Residual Disease After Ixazomib Plus Lenalidomide Plus Dexamethasone (IRd) Treatment for Newly Diagnosed Transplant Eligible Myeloma Patients (Silvennoinen et al., 2023)^49^ | NMDD | N | Ph2 | See comment. | NA | 120 | NA | Minimal Residual Disease | N |
| A Phase 1/2 Study of MLN9708 (Ixazomib [I]), Venetoclax (V), and Dexamethasone (D) Regimen (IVD) to Restore or Enhance Proteasome Inhibitor (PI) Sensitivity in Non-t(11;14) Relapsed/Refractory Multiple Myeloma (RRMM) (NCT03856112) | R/R | Y | Ph1/2 | Experimental: Arm I (ixazomib, venetoclax, dexamethasone)  Experimental: Arm III (ixazomib, venetoclax, dexamethasone) | Active Comparator: Arm II (ixazomib, dexamethasone) | 0 | NA | Safety, Response Rate | Cannot be ascertained |
| A Multicenter, Open Label, Randomized Phase II Study Comparing Daratumumab Combined With Bortezomib-Cyclophosphamide-dexamethasone (Dara-VCd) Versus the Association of Bortezomib-Thalidomide-dexamethasone (VTd) as Pre Transplant Induction and Post Transplant (NCT03896737) | NMDD, maintenance | Y | Ph2 | Maintenance: Ixazomib + Dartumumab | Maintenance: Ixazomib | NA | NA | Progression Free Survival, Minimal Residual Disease | Cannot be ascertained |
| Optimizing Prolonged Treatment In Myeloma Using MRD Assessment (OPTIMUM) (NCT03941860) | maintenance | Y | Ph3 | Ixazomib + Lenalidomide | Lenalidomide + Placebo | Study had 1 participant that eventually withdrew | NA | Overall Survival | Cannot be ascertained |
| A Randomized Phase II Study of Daratumumab, Ixazomib, and Dexamethasone (DId, Arm A) Vs Daratumumab, Bortezomib and Dexamethasone (DVd) Followed By Daratumumab, Did (Arm B) in Newly Diagnosed Multiple Myeloma (DeRIVE) Study (Nooka et al., 2023)^50^ | NMDD, maintenance | Y | Ph2 | Arm I (DId): Induction (8 cycles, 28-day cycle):  Daratumumab (IV): Days 1, 8, 15, 22 (cycles 1–2), then Days 1, 15 (cycles 3–8)  Ixazomib (PO): Days 1, 8, 15  Dexamethasone (IV/PO): Days 1, 8, 15, 22  Maintenance (up to 24 months):  Daratumumab (IV): Day 1 of each 28-day cycle  Ixazomib (PO): Days 1, 8, 15  Dexamethasone (IV/PO): Days 1, 8, 15, 22 | Arm II (DVd → DId): Induction Cycles 1–3 (21-day cycles):  Daratumumab (IV): Days 1, 8, 15  Bortezomib (SC): Days 1, 4, 8, 11  Dexamethasone (IV/PO): Days 1, 8, 15  Induction Cycles 4–8 (28-day cycles):  Switch to DId (same as Arm I)  Maintenance (up to 24 months):  Same as Arm I | 25 | 23 | Response Rate | Cannot be ascertained |
| Phase II Open Lable Clinical Study Efficacy and Safety of the Holistic Treatment for Young Patients With High-Risk Multiple Myeloma (NCT04008888) | NMDD, R/R, maintenance | N | N/A | Arm A: Fludarabine+Melphalan followed by Allogeneic SCT Arm B: Melphalan followed by Autologous SCT Both receive Ixazomib OR Bortezomib and dexamethasone for maintenance | Control: Consolidated Chemotherapy for Patients Unable to Receive Transplantation Will receive Ixazomib OR Bortezomib + Dexamethasone for maintenance | NA | NA | Progression Free Survival | Cannot be ascertained |
| A Phase II Study of Lenalidomide, Ixazomib, Dexamethasone, and Daratumumab in Transplant-Ineligible Patients With Newly Diagnosed Multiple Myeloma (O’Donnell et al., 2021)^51^ | NMDD, maintenance | Y | Ph2 | Both arms go through inducation with: 12 cycles of ixazomib + lenalidomide + dexamethasone + daratumumab (IRd-D); Maintenance:  Arm A: Lenalidomide alone (until progression/unacceptable toxicity or 2 years)  Arm B: Lenalidomide + ixazomib + daratumumab (same duration criteria) | Lenalidomide | 94 | 94 | Progression Free Survival | Cannot be ascertained |
| A Phase 2 Study of Subcutaneous Daratumumab in Combination With Dose-Attenuated Bortezomib, Lenalidomide, and Dexamethasone in Elderly Newly Diagnosed Multiple Myeloma Patients (NCT04052880) | NMDD | N | Ph2 | Daratumumab, Bortezomib, Lenalidomide, Dexamethasone, Ixazomib | NA | 20 | NA | Response Rate | Cannot be ascertained |
| Phase I/II Study of Twice Weekly Ixazomib Plus Pomalidomide and Dexamethasone in Relapsed/or Refractory Multiple Myeloma (Nadeem et al., 2022)^52^ | R/R | N | Ph1/2 | ixazomib plus pomalidomide and dexamethasone | NA | 30 | NA | Response Rate, Safety | Cannot be ascertained |
| A Phase II Study of Nivolumab in Combination With Ixazomib, Cyclophosphamide, and Dexamethasone in Relapsed and Refractory Multiple Myeloma (NCT04119336) | R/R | N | Ph2 | Nivolumab + Ixazomib | NA | 2 | NA | Response Rate | Cannot be ascertained |
| Ixazomib versus Lenalidomide or Ixazomib and Lenalidomide combination as maintenance regimen for patients with multiple myeloma: interim analysis of a multi-center prospective study in China (Zhuang et al., 2022)^53^ | maintenance | N | Ph4 | Experimental Arm: Ixazomib + Lenalidomide Comparator Arm 1: Lenalidomide Comparator Arm 2: Ixazomib | NA | 211 | NA | Progression Free Survival, Overall Survival | Cannot be ascertained |
| A Phase 1 Study of MLN9708 in Japanese Patients With Relapsed and/or Refractory Multiple Myeloma (NCT04272775) | R/R | N | Ph1 | Ixazomib | Ixazomib 4.0 mg + Lenalidomide and Dexamethasone | 7 | 7 | Safety | Cannot be ascertained |
| Phase 1 Study of Tasquinimod Alone and in Combination with Standard Therapy for Relapsed or Refractory Myeloma (Vogl et al., 2020)^54^ | R/R | N | Ph1 | Tasquinimod + Ixazomib + Lenalidomide + Dexamethasone | Tasquinimod (single agent) | 12 | NA | Safety | Cannot be ascertained |
| A Phase II Multicenter Clinical Trial Assessing the Safety and Efficacy of Ixazomib Pomalidomide Dexamethasone Combination As Second or Third-Line Treatment for Patients with Triple Exposed Relapsed and Refractory Multiple Myeloma: Prespecified Interim Analysis (IPoD-790 trial) (Cohen et al., 2022)^55^ | R/R | N | Ph2 | ixazomib-pomalidomide-dexamethasone | NA | 22 | NA | Progression Free Survival | Cannot be ascertained |
| A Multi-center, Open-label, Single-arm Clinical Study of Ixazomib/Pomalidomide/Dexamethasone (IxaPD) in the Treatment of Patients With Relapsed Multiple Myeloma (Chen *et al.*)^56^ | R/R | N | Ph4 | Ixazomib, pomalidomide, dexamethasone | NA | 60 | NA | Progression Free Survival |  |
| A Multicenter In-class Transition Study of Ixazomib Combined With Pomalidomide and Dexamethasone or With Lenalidomide and Dexamethasone in Adults With Relapsed/Refractory Multiple Myeloma (US MM-7) (NCT05183139) | R/R | N | Ph4 | Two experimental arms, Arm A: Ixazomib 4 mg + Pomalidomide 4 mg + Dexamethasone 40 mg; Arm B: Ixazomib 4 mg + Lenalidomide 25 mg + Dexamethasone 40 mg | NA | NA | NA | Progression Free Survival | Cannot be ascertained |
| Id and Rd Maintenance Regimens After Induction of Remission in Multiple Myeloma: a Prospective, Randomized, Controlled, Multicenter Clinical Study (NCT05477797) | maintenance | Y | N/A | Ixazomib + Dexamethasone | Lenalidomide + Dexamethasone | NA | NA | Progression Free Survival | Cannot be ascertained |
| Ixazomib Plus Low-dose Lenalidomide Versus Ixazomib Alone for Maintenance Treatment of High Risk Multiple Myeloma (NCT05722405) | maintenance | Y | Ph4 | Ixazomid + Low Dose Lenalidomide | Ixazomib | NA | NA | Progression Free Survival | Cannot be ascertained |
| Phase I/II Trial of Mezigdomide Plus Ixazomib and Dexamethasone for Relapsed and Refractory Multiple Myeloma (NCT06050512) | R/R | N | Ph1/2 | Mezigdomide + Ixazomib + Dexamethasone | NA | NA | NA | Response Rate, Safety | Cannot be ascertained |
| UARK 2015-03 A Phase II Trial of a Novel Proteasome/IMiD Combination (NCT02578121) | R/R | N | Ph2 | Ixazomib, Pomalidomide, Dexamethasone | NA | NA | NA | Response Rate | Cannot be ascertained |
| A MULTIARM, OPEN LABEL, RANDOMIZED PHASE II STUDY OF MLN9708 PLUS ORAL DEXAMETHASONE or PLUS ORAL CYCLOPHOSPHAMIDE AND DEXAMETHASONE or PLUS ORAL THALIDOMIDE AND DEXAMETHASONE FOLLOWED BY MAINTENANCE WITH MLN9708 IN NEWLY DIAGNOSED ELDERLY MULTIPLE MYELOMA (NCT02586038) | NMDD | Y | Ph2 | Ixazomib, Dexamethasone, Cyclophosphamide | Ixazomib, Dexamethasone, Thalidomide | NA | NA | Overall Survival | Cannot be ascertained |
| Phase I Study of Selinexor, Ixazomib, and Low-dose Dexamethasone in Patients With Relapsed or Refractory Multiple Myeloma (Salcedo et al., 2020)^45^ | R/R | N | Ph1 | Selinexor (KPT-330), Ixazomib, and Dexamethasone | NA | NA | NA | Safety | Cannot be ascertained |
| Phase I Clinical Trial of Venetoclax (ABT-199) in Combination with Ixazomib and Dexamethasone for Patients with Relapsed Multiple Myeloma (NCT03399539) | R/R | N | Ph1 | (venetoclax, ixazomib citrate, dexamethasone) | NA | NA | NA | Progression Free Survival, Safety, Overall Survival, Response Rate | Cannot be ascertained |
| A Phase I/II Study of the Addition of Ixazomib to ONC201 and Dexamethasone in Relapsed and/or Refractory Multiple Myeloma (NCT03492138) | R/R | N | Ph1 | ONC201, ixazomib, and dexamethasone | NA | NA | NA | Safety | Cannot be ascertained |
| Feasibility/Phase II Study of Myeloablative BEAM Allogeneic Transplantation Followed by Oral Ixazomib Maintenance Therapy in Patients With High-Risk Myeloma (Emma-Scott et al., 2017) ^57^ | R/R | N | Ph1 | Carmustine, Cytarabine, Etoposide, Melphalan, Tacrolimus, Methotrexate, Ixazomib | NA | 6 | NA | Safety | Cannot be ascertained |
| Toward a Risk-adapted Strategy to Cure Myeloma : An Intensive Program With Lenalidomide, Ixazomib, and Dexamethasone Plus Daratumumab as Extended Induction and Consolidation Followed by Lenalidomide Maintenance in Newly Diagnosed Standard Risk Multiple Myeloma (NCT03669445) | NMDD | N | Ph2 | Ixazomib, Lenalidomide, Dexamethasone, Daratumumab | NA | NA | NA | Minimal Residual Disease | Cannot be ascertained |
| Frailty-adjusted therapy in Transplant Non-Eligible patients with newly diagnosed Multiple Myeloma (FiTNEss (UK-MRA Myeloma XIV Trial)): a study protocol for a randomised phase III trial (Coulson et al., 2022)^58^ | NMDD, maintenance | Y | Ph3 | R1: At R1, eligible patients will be allocated to one of two interventions with IRD induction; standard up-front dosing followed by toxicity dependent dose modification (reactive), or; frailty score-adjusted up-front dose reductions (adaptive).  R2: At R2, eligible patients will be randomised between lenalidomide+ixazomib (R+I) or lenalidomide and placebo maintenance therapy. | Ixazomib | NA | NA | Safety | Cannot be ascertained |
| Pomalidomide, Ixazomib, and Dexamethasone (PId) With or Without Intensification by Cyclophosphamide (PICd): A Phase II Study in Relapsed or Refractory Multiple Myeloma (NCT03731832) | R/R | N | Ph2 | Pomalidomide, Ixazomib, Dexamethasone | NA | NA | NA | Response Rate | Cannot be ascertained |
| Myeloma Developing Regimens Using Genomics (MyDRUG): Longitudinal Single-Cell Transcriptional Landscape of the Myeloma and Immune Microenvironment in Relapsed/Refractory Multiple Myeloma Patients Treated with MEK-Inhibitor, Cobimetinib (Jayasinghe et al., 2022)^59^ | R/R | N | Ph1/2 | Complex. See comment | Complex. See comment | 9 | NA | Response Rate | Cannot be ascertained |
| A Phase 2, Open Label, Randomized Trial Evaluating Ixazomib Compared to Ixazomib-Lenalidomide Combination Maintenance Therapy for Frontline Multiple Myeloma Patients (NCT03733691) | maintenance | Y | Ph2 | Ixazomib + Lenalidomide | Ixazomib | NA | NA | Progression Free Survival, Safety | Cannot be ascertained |
| A Multicentre Open-label Phase II Study of Ixazomib -Daratumumab Without Dexamethasone (IDara) in Elderly Relapse Refractory Multiple Myeloma (NCT03757221) | R/R | N | Ph2 | Ixazomib + daratumumab | NA | NA | NA | Response Rate | Cannot be ascertained |
| Initial Clinical Results and Immune Correlates of a Phase 2 Study of Daratumumab, Bortezomib, Dexamethasone Followed By a Proteasome Inhibitor in-Class Transition ( iCT) to Daratumumab, Ixazomib, Dexamethasone in Relapsed Refractory Multiple Myeloma (Lee et al., 2023)^60^ | R/R | N | Ph2 | daratumumab, bortezomib, dexamethasone, ixazomib | NA | 36 | NA | Progression Free Survival | Cannot be ascertained |

NMDD: Newly Diagnosed Multiple Myeloma

R/R: Relapsed/refractory

Ph: Phase

Y: Yes

N: No

1.	Li S, Zhang D, Yang L, Huang C, Niu T, Gong Y. Comparison between ixazomib+cyclophosphamide+dexamethasone regimen and ixazomib+dexamethasone regimen for elderly and frail patients having newly diagnosed multiple myeloma. *Cancer Med* 2023; **12**(6): 6523–35.

2.	Richardson PG, Kumar SK, Masszi T, et al. Final Overall Survival Analysis of the TOURMALINE-MM1 Phase III Trial of Ixazomib, Lenalidomide, and Dexamethasone in Patients With Relapsed or Refractory Multiple Myeloma. *J Clin Oncol* 2021; **39**(22): 2430–42.

3.	Wang Y, Liu YF, Jin SW, et al. Ixazomib or Lenalidomide combined with cyclophosphamide and dexamethasone in the treatment of elderly transplant-ineligible newly diagnosed multiple myeloma. *Sci Rep* 2025; **15**(1): 6538.

4.	Cook G, Ashcroft AJ, Senior E, et al. Ixazomib as consolidation and maintenance versus observation in patients with relapsed multiple myeloma eligible for salvage autologous stem-cell transplantation (Myeloma XII [ACCoRD]): interim analysis of a multicentre, open-label, randomised, phase 3 trial. *Lancet Haematol* 2024; **11**(11): e816–e29.

5.	Auner HW, Brown SR, Walker K, et al. Ixazomib with cyclophosphamide and dexamethasone in relapsed or refractory myeloma: MUKeight phase II randomised controlled trial results. *Blood Cancer J* 2022; **12**(4): 52.

6.	Richardson PG, Baz R, Wang M, et al. Phase 1 study of twice-weekly ixazomib, an oral proteasome inhibitor, in relapsed/refractory multiple myeloma patients. *Blood* 2014; **124**(7): 1038–46.

7.	Kumar SK, Bensinger WI, Zimmerman TM, et al. Phase 1 study of weekly dosing with the investigational oral proteasome inhibitor ixazomib in relapsed/refractory multiple myeloma. *Blood* 2014; **124**(7): 1047–55.

8.	Kumar SK, Berdeja JG, Niesvizky R, et al. Ixazomib, lenalidomide, and dexamethasone in patients with newly diagnosed multiple myeloma: long-term follow-up including ixazomib maintenance. *Leukemia* 2019; **33**(7): 1736–46.

9.	San-Miguel JF, Echeveste Gutierrez MA, Špicka I, et al. A phase I/II dose-escalation study investigating all-oral ixazomib-melphalan-prednisone induction followed by single-agent ixazomib maintenance in transplant-ineligible newly diagnosed multiple myeloma. *Haematologica* 2018; **103**(9): 1518–26.

10.	Richardson PG, Hofmeister CC, Rosenbaum CA, et al. Twice-weekly ixazomib in combination with lenalidomide-dexamethasone in patients with newly diagnosed multiple myeloma. *Br J Haematol* 2018; **182**(2): 231–44.

11.	Kumar SK, LaPlant BR, Reeder CB, et al. Randomized phase 2 trial of ixazomib and dexamethasone in relapsed multiple myeloma not refractory to bortezomib. *Blood* 2016; **128**(20): 2415–22.

12.	Gupta N, Goh YT, Min CK, et al. Pharmacokinetics and safety of ixazomib plus lenalidomide-dexamethasone in Asian patients with relapsed/refractory myeloma: a phase 1 study. *J Hematol Oncol* 2015; **8**: 103.

13.	Shah JJ, Baladandayuthapani V, Weber DM, et al. Phase II Study Of The Combination Of MLN 9708 With Lenalidomide As Maintenance Therapy Post Autologous Stem Cell Transplant In Patients With Multiple Myeloma. *Blood* 2013; **122**(21): 1983–.

14.	Gupta N, Hanley MJ, Harvey RD, et al. A pharmacokinetics and safety phase 1/1b study of oral ixazomib in patients with multiple myeloma and severe renal impairment or end-stage renal disease requiring haemodialysis. *Br J Haematol* 2016; **174**(5): 748–59.

15.	Facon T, Venner CP, Bahlis NJ, et al. Oral ixazomib, lenalidomide, and dexamethasone for transplant-ineligible patients with newly diagnosed multiple myeloma. *Blood* 2021; **137**(26): 3616–28.

16.	Kumar SK, Buadi FK, LaPlant B, et al. Phase 1/2 trial of ixazomib, cyclophosphamide and dexamethasone in patients with previously untreated symptomatic multiple myeloma. *Blood Cancer J* 2018; **8**(8): 70.

17.	Touzeau C, Perrot A, Roussel M, et al. All-oral triplet combination of ixazomib, lenalidomide, and dexamethasone in newly diagnosed transplant-eligible multiple myeloma patients: final results of the phase II IFM 2013-06 study. *Haematologica* 2022; **107**(7): 1693–7.

18.	Voorhees P, Suman V, Efebera Y, et al. Alliance A061202: ixazomib, pomalidomide, and dexamethasone for patients with lenalidomide-refractory MM in first relapse. *Blood Adv* 2024; **8**(19): 5039–50.

19.	Kumar SK, Grzasko N, Delimpasi S, et al. Phase 2 study of all-oral ixazomib, cyclophosphamide and low-dose dexamethasone for relapsed/refractory multiple myeloma. *Br J Haematol* 2019; **184**(4): 536–46.

20.	Reu FJ, Valent J, Malek E, et al. A Phase I Study of Ixazomib in Combination with Panobinostat and Dexamethasone in Patients with Relapsed or Refractory Multiple Myeloma. *Blood* 2015; **126**(23): 4221–.

21.	Krishnan A, Kapoor P, Palmer JM, et al. Phase I/II trial of the oral regimen ixazomib, pomalidomide, and dexamethasone in relapsed/refractory multiple myeloma. *Leukemia* 2018; **32**(7): 1567–74.

22.	Bachier CR, Shaughnessy PJ, Matous J, Berdeja JG. 339 - Open-Label Study to Determine the Feasibility of Ixazomib as Maintenance after Allogeneic Stem Cell Transplant for Multiple Myeloma. *Biology of Blood and Marrow Transplantation* 2018; **24**(3, Supplement): S259.

23.	Goldschmidt H, Dimopoulos MA, Rajkumar SV, et al. Deepening responses associated with improved progression-free survival with ixazomib versus placebo as posttransplant maintenance in multiple myeloma. *Leukemia* 2020; **34**(11): 3019–27.

24.	Daniely D, Forouzan E, Spektor TM, et al. A phase 1/2 study of ixazomib in place of bortezomib or carfilzomib in a subsequent line of therapy for patients with multiple myeloma refractory to their last bortezomib or carfilzomib combination regimen. *Exp Hematol* 2022; **111**: 79–86.

25.	Slade M, Martin TG, Nathwani N, et al. Ixazomib, lenalidomide and dexamethasone consolidation with randomized ixazomib or lenalidomide maintenance after autologous transplant in newly diagnosed multiple myeloma. *Leukemia* 2022; **36**(12): 2917–21.

26.	Dimopoulos MA, Špička I, Quach H, et al. Ixazomib as Postinduction Maintenance for Patients With Newly Diagnosed Multiple Myeloma Not Undergoing Autologous Stem Cell Transplantation: The Phase III TOURMALINE-MM4 Trial. *J Clin Oncol* 2020; **38**(34): 4030–41.

27.	Rosiñol L, Oriol A, Ríos R, et al. Lenalidomide and dexamethasone maintenance with or without ixazomib, tailored by residual disease status in myeloma. *Blood* 2023; **142**(18): 1518–28.

28.	Ludwig H, Poenisch W, Knop S, et al. Ixazomib-Thalidomide-Dexamethasone for induction therapy followed by Ixazomib maintenance treatment in patients with relapsed/refractory multiple myeloma. *Br J Cancer* 2019; **121**(9): 751–7.

29.	Pelcovits A, Barth P, Reagan JL, et al. Ixazomib, Oral Metronomic Cyclophosphamide, and Dexamethasone for First-Line Treatment of Multiple Myeloma: A Phase II Brown University Oncology Group Study. *Oncologist* 2023; **28**(5): 462–e303.

30.	Bashir Q, Nishihori T, Pasquini MC, et al. A Multicenter Phase II, Double-Blind, Placebo-Controlled Trial of Maintenance Ixazomib After Allogeneic Transplantation for High-Risk Multiple Myeloma: Results of the Blood and Marrow Transplant Clinical Trials Network 1302 Trial. *Transplant Cell Ther* 2023; **29**(6): 358.e1–.e7.

31.	Dhakal B, D'Souza A, Hamadani M, et al. Phase I/II trial of bendamustine, ixazomib, and dexamethasone in relapsed/refractory multiple myeloma. *Blood Cancer J* 2019; **9**(8): 56.

32.	Buadi FK, Lacy MQ, Perez G, et al. Phase 2 Trial of Pomalidomide, Ixazomib and Dexamethasone in Patients with Multiple Myeloma with Extramedullary Disease or Plasma Cell Leukemia. *Blood* 2020; **136**(Supplement 1): 34–5.

33.	Mina R, Falcone AP, Bringhen S, et al. Ixazomib-based induction regimens plus ixazomib maintenance in transplant-ineligible, newly diagnosed multiple myeloma: the phase II, multi-arm, randomized UNITO-EMN10 trial. *Blood Cancer J* 2021; **11**(12): 197.

34.	Holmberg LA, Green DJ, Lee SS, Cowan AJ. Alternating the Administration of Ixazomib and Lenalidomide As Maintenance Therapy after Autologous Transplant for Multiple Myeloma. *Blood* 2022; **140**(Supplement 1): 10575–6.

35.	Bernal-Mizrachi L, Cole CE, Heffner L, et al. Phase II Trial of Ixazomib and Dexamethasone Versus Ixazomib, Dexamethasone and Lenalidomide, Randomized with NFKB2 Rearrangement. (Proteasome Inhibitor NFKB2 Rearrangement Driven Trial, PINR). *Blood* 2018; **132**(Supplement 1): 2011–.

36.	Iida S, Izumi T, Komeno T, et al. A phase 2, open-label, multicenter study of ixazomib plus lenalidomide and dexamethasone in adult Japanese patients with relapsed and/or refractory multiple myeloma. *Int J Clin Oncol* 2022; **27**(1): 224–33.

37.	Kumar SK, Kapoor P, Laplant B, et al. Phase 2 Trial of Ixazomib, Lenalidomide, Dexamethasone and Daratumumab in Patients with Newly Diagnosed Multiple Myeloma. *Blood* 2018; **132**(Supplement 1): 304–.

38.	Abe Y, Sasaki M, Takezako N, et al. Efficacy and Safety of Ixazomib Plus Lenalidomide and Dexamethasone Following Injectable PI-Based Therapy in Relapsed/Refractory Multiple Myeloma. *Ann Hematol* 2023; **102**(9): 2493–504.

39.	Delimpasi S, Dimopoulos MA, Straub J, et al. Ixazomib plus daratumumab and dexamethasone: Final analysis of a phase 2 study among patients with relapsed/refractory multiple myeloma. *Am J Hematol* 2024; **99**(9): 1746–56.

40.	Fu W, Du J, Zhong L, et al. Ixazomib Maintenance Therapy in Chinese Patients with Newly Diagnosed Multiple Myeloma Not Treated with Stem Cell Transplantation: A Single-Arm, Open-Label Study. *Blood* 2023; **142**(Supplement 1): 6735–.

41.	Touzeau C, Leleu X, Tiab M, et al. Iberdomide, ixazomib and dexamethasone in elderly patients with multiple myeloma at first relapse. *Br J Haematol* 2025; **206**(5): 1366–72.

42.	Stege CAM, Nasserinejad K, van der Spek E, et al. Ixazomib, Daratumumab, and Low-Dose Dexamethasone in Frail Patients With Newly Diagnosed Multiple Myeloma: The Hovon 143 Study. *J Clin Oncol* 2021; **39**(25): 2758–67.

43.	Zweegman S, Stege CAM, Haukas E, et al. Ixazomib-Thalidomide-low dose dexamethasone induction followed by maintenance therapy with ixazomib or placebo in newly diagnosed multiple myeloma patients not eligible for autologous stem cell transplantation; results from the randomized phase II HOVON-126/NMSG 21.13 trial. *Haematologica* 2020; **105**(12): 2879–82.

44.	Bergin K, Yuen F, Wallington-Beddoe C, et al. A phase II trial of continuous ixazomib, thalidomide and dexamethasone for relapsed and/or refractory multiple myeloma: the Australasian Myeloma Research Consortium (AMaRC) 16-02 trial. *Br J Haematol* 2021; **194**(3): 580–6.

45.	Salcedo M, Lendvai N, Mastey D, et al. Phase I Study of Selinexor, Ixazomib, and Low-dose Dexamethasone in Patients With Relapsed or Refractory Multiple Myeloma. *Clin Lymphoma Myeloma Leuk* 2020; **20**(3): 198–200.

46.	Dimopoulos MA, Schjesvold F, Doronin V, et al. Oral ixazomib-dexamethasone vs oral pomalidomide-dexamethasone for lenalidomide-refractory, proteasome inhibitor-exposed multiple myeloma: a randomized Phase 2 trial. *Blood Cancer J* 2022; **12**(1): 9.

47.	Terpos E, Ntanasis-Stathopoulos I, Gavriatopoulou M, et al. Efficacy and safety of daratumumab with ixazomib and dexamethasone in lenalidomide-exposed patients after one prior line of therapy: Final results of the phase 2 study DARIA. *Am J Hematol* 2024; **99**(3): 396–407.

48.	Diaz-delCastillo M, Gundesen MT, Andersen CW, et al. Increased Bone Volume by Ixazomib in Multiple Myeloma: 3-Month Results from an Open Label Phase 2 Study. *J Bone Miner Res* 2023; **38**(5): 639–49.

49.	Silvennoinen R, Partanen A, Waage A, et al. P898: A Prospective Phase 2 Study to Asess Minimal Residual Disease After Ixazomib, Lenalidomide, Dexamethasone (IRD) Treatment for Newly Diagnosed Transplant Eligible Multiple Myeloma Patients. *Hemasphere* 2023; **7**(Suppl).

50.	Nooka AK, Joseph NS, Gupta VA, et al. A Randomized Phase II Study of Daratumumab, Ixazomib, and Dexamethasone (DId, Arm A) Vs Daratumumab, Bortezomib and Dexamethasone (DVd) Followed By Daratumumab, Did (Arm B) in Newly Diagnosed Multiple Myeloma (DeRIVE) Study. *Blood* 2023; **142**(Supplement 1): 4764–.

51.	O'Donnell EK, Suman VJ, Geyer SM, et al. A Phase II Study of Lenalidomide, Ixazomib, Dexamethasone, and Daratumumab in Transplant-Ineligible Patients with Newly Diagnosed Multiple Myeloma (AFT-41). *Blood* 2021; **138**(Supplement 1): 4776–.

52.	Nadeem O, Redd RA, Bayliss TJ, et al. A Phase I/II Study of Twice Weekly Ixazomib Plus Pomalidomide and Dexamethasone in Relapsed and Refractory Multiple Myeloma. *Blood* 2022; **140**(Supplement 1): 10180–1.

53.	Zhuang Z, Tian Y, Yu H, et al. P978: Ixazomib Versus Lenalidomide or Ixazomib and Lenalidomide Combination as Maintenance Regimen for Patients with Multiple Myeloma: Interim Analysis of a Multi-center Prospective Study in China. *Hemasphere* 2022; **6**(Suppl).

54.	Vogl DT, Nefedova Y, Wileyto EP, et al. A Phase 1 Study of Tasquinimod in Patients with Relapsed or Refractory Multiple Myeloma. *Blood* 2020; **136**(Supplement 1): 17–8.

55.	Cohen YC, Shragai T, Lavi N, et al. A Phase II Multicenter Clinical Trial Assessing the Safety and Efficacy of Ixazomib Pomalidomide Dexamethasone Combination As Second or Third-Line Treatment for Patients with Triple Exposed Relapsed and Refractory Multiple Myeloma: Prespecified Interim Analysis (IPoD-790 trial). *Blood* 2022; **140**(Supplement 1): 10131–2.

56.	Chen M, Bao L, Chen W, et al. Trial in Progress: A Perspective Study of Pan-Oral Triplet Regimen Pomalidomide, Ixazomib and Dexamethasone in Relapsed or Refractory Myeloma Patients. *Blood* 2021; **138**(Supplement 1): 4783–.

57.	Scott RM, Dreumont S, Gasparetto C. Ixazomib Versus Lenalidomide or Ixazomib and Lenalidomide Combination as Maintenance Regimen for Patients with Multiple Myeloma: Interim Analysis of a Multi-center Prospective Study in China. Hemasphere 2017; 1(Suppl).

58.	Coulson AB, Royle KL, Pawlyn C, et al. Frailty-adjusted therapy in Transplant Non-Eligible patients with newly diagnosed Multiple Myeloma (FiTNEss (UK-MRA Myeloma XIV Trial)): a study protocol for a randomised phase III trial. *BMJ Open* 2022; **12**(6): e056147.

59.	Jayasinghe RG, Mulligan G, Cook A, et al. Myeloma Developing Regimens Using Genomics (MyDRUG): Longitudinal Single-Cell Transcriptional Landscape of the Myeloma and Immune Microenvironment in Relapsed/Refractory Multiple Myeloma Patients Treated with MEK-Inhibitor, Cobimetinib. *Blood* 2022; **140**(Supplement 1): 7090–1.

60.	Lee HC, Li J, Lin H, et al. Initial Clinical Results and Immune Correlates of a Phase 2 Study of Daratumumab, Bortezomib, Dexamethasone Followed By a Proteasome Inhibitor in-Class Transition ( iCT) to Daratumumab, Ixazomib, Dexamethasone in Relapsed Refractory Multiple Myeloma. *Blood* 2023; **142**(Supplement 1): 4760–.
