# Supplementary material for: Clinical trials portfolio and regulatory outcomes of ixazomib in multiple myeloma: A systematic review and meta‐analysis
Source: Br J Haematol. 2026 Aug 23;209(4):1882–5. doi: 10.1111/bjh.70757 (PMC13674204; doi:10.1111/bjh.70757)
Supplement: Supplementary file 2 [file BJH-209-1882-s003.docx]

**Supplementary Table 2. TOURMALINE-MM series of large randomized clinical trials**

| **Trial** | **Trial population** | **Intervention Arm** | **Control Arm** | **Results** |
| --- | --- | --- | --- | --- |
| **MM1** | Patients with refractory or relapsed myeloma that had progressed following 1-3 previous therapies | Ixazomib + Len + dex (n=361) | Placebo + Len + Dex (n=359) | Statistically significant improvement in PFS (HR: 0.74 [95% CI, 0.59–0.94], p=0.01), no difference in OS (median 53.6 vs 51.6 months, HR: 0.939, p= 0.495) |
| **MM2** | Patients with newly diagnosed myeloma | Ixazomib + Len + Dex Combined (n=354) | Placebo + Len + Dex Combined (n=349) | No statistically significant benefit in either PFS or OS |
| **MM3** | Patients with newly diagnosed myeloma following autologous stem cell transplant | Ixazomib Citrate (n=394) | Placebo (n=259) | PFS benefits (HR: 0.72 [95% CI 0.58–0.89], p=0.0023) in the ixazomib arm |
| **MM4** | Patients with newly diagnosed myeloma ineligible for transplant | Ixazomib (n=426) | Placebo (n=276) | Improvement in PFS (HR: 0.659 [95% CI 0.542-0.801], p<0.001) compared to the placebo |

MM2 Note: Combined "Up to 18 Cycles" + "≥19 Cycles" (Placebo: 160+189=349, Ixazomib: 163+191=354)
Len: lenalidomide, Dex: dexamethasone

**Supplementary Table 3: Serious Adverse Events that are Consistently Reported Across All 4 TOURMALINE Clinical Trials. Source:** clinicaltrials.gov

| **Adverse Event** | **Category** | **MM1 Ixa** | **MM1 Placebo** | **MM2 Ixa** | **MM2 Placebo** | **MM3 Ixa** | **MM3 Placebo** | **MM4 Ixa** | **MM4 Placebo** |
| --- | --- | --- | --- | --- | --- | --- | --- | --- | --- |
| **Blood and lymphatic system disorders (1 events)** | | | | | | | | | |
| Anaemia | Blood and lymphatic system disorders | 5/361 (1.39%) | 10/359 (2.79%) | 7/354 (1.98%) | 4/349 (1.15%) | 0/394 (0.00%) | 1/259 (0.39%) | 0/426 (0.00%) | 3/276 (1.09%) |
| **Cardiac disorders (2 events)** | | | | | | | | | |
| Cardiac failure | Cardiac disorders | 4/361 (1.11%) | 6/359 (1.67%) | 5/354 (1.41%) | 2/349 (0.57%) | 1/394 (0.25%) | 0/259 (0.00%) | 1/426 (0.23%) | 0/276 (0.00%) |
| Myocardial infarction | Cardiac disorders | 5/361 (1.39%) | 2/359 (0.56%) | 0/354 (0.00%) | 3/349 (0.86%) | 0/394 (0.00%) | 1/259 (0.39%) | 0/426 (0.00%) | 2/276 (0.72%) |
| **Gastrointestinal disorders (4 events)** | | | | | | | | | |
| Abdominal pain upper | Gastrointestinal disorders | 1/361 (0.28%) | 0/359 (0.00%) | 1/354 (0.28%) | 0/349 (0.00%) | 0/394 (0.00%) | 1/259 (0.39%) | 1/426 (0.23%) | 0/276 (0.00%) |
| Diarrhea | Gastrointestinal disorders | 12/361 (3.32%) | 3/359 (0.84%) | 15/354 (4.24%) | 1/349 (0.29%) | 4/394 (1.02%) | 0/259 (0.00%) | 2/426 (0.47%) | 0/276 (0.00%) |
| Inguinal hernia | Gastrointestinal disorders | 3/361 (0.83%) | 2/359 (0.56%) | 0/354 (0.00%) | 2/349 (0.57%) | 1/394 (0.25%) | 0/259 (0.00%) | 3/426 (0.70%) | 0/276 (0.00%) |
| Vomiting | Gastrointestinal disorders | 2/361 (0.55%) | 0/359 (0.00%) | 2/354 (0.56%) | 1/349 (0.29%) | 1/394 (0.25%) | 0/259 (0.00%) | 3/426 (0.70%) | 2/276 (0.72%) |
| **General disorders (2 events)** | | | | | | | | | |
| Non-cardiac chest pain | General disorders | 2/361 (0.55%) | 1/359 (0.28%) | 2/354 (0.56%) | 1/349 (0.29%) | 0/394 (0.00%) | 1/259 (0.39%) | 2/426 (0.47%) | 0/276 (0.00%) |
| Pyrexia | General disorders | 12/361 (3.32%) | 18/359 (5.01%) | 7/354 (1.98%) | 6/349 (1.72%) | 5/394 (1.27%) | 2/259 (0.77%) | 2/426 (0.47%) | 1/276 (0.36%) |
| **Infections and infestations (10 events)** | | | | | | | | | |
| Bronchitis | Infections and infestations | 6/361 (1.66%) | 10/359 (2.79%) | 7/354 (1.98%) | 5/349 (1.43%) | 3/394 (0.76%) | 0/259 (0.00%) | 1/426 (0.23%) | 0/276 (0.00%) |
| Cellulitis | Infections and infestations | 4/361 (1.11%) | 2/359 (0.56%) | 3/354 (0.85%) | 4/349 (1.15%) | 2/394 (0.51%) | 0/259 (0.00%) | 0/426 (0.00%) | 1/276 (0.36%) |
| Gastroenteritis | Infections and infestations | 5/361 (1.39%) | 1/359 (0.28%) | 5/354 (1.41%) | 3/349 (0.86%) | 2/394 (0.51%) | 1/259 (0.39%) | 0/426 (0.00%) | 1/276 (0.36%) |
| Herpes zoster | Infections and infestations | 3/361 (0.83%) | 3/359 (0.84%) | 3/354 (0.85%) | 0/349 (0.00%) | 4/394 (1.02%) | 2/259 (0.77%) | 1/426 (0.23%) | 0/276 (0.00%) |
| Influenza | Infections and infestations | 8/361 (2.22%) | 5/359 (1.39%) | 3/354 (0.85%) | 6/349 (1.72%) | 4/394 (1.02%) | 1/259 (0.39%) | 0/426 (0.00%) | 1/276 (0.36%) |
| Lower respiratory tract infection | Infections and infestations | 6/361 (1.66%) | 4/359 (1.11%) | 1/354 (0.28%) | 2/349 (0.57%) | 3/394 (0.76%) | 1/259 (0.39%) | 5/426 (1.17%) | 1/276 (0.36%) |
| Pneumonia | Infections and infestations | 47/361 (13.02%) | 46/359 (12.81%) | 44/354 (12.43%) | 29/349 (8.31%) | 25/394 (6.35%) | 10/259 (3.86%) | 16/426 (3.76%) | 2/276 (0.72%) |
| Respiratory tract infection | Infections and infestations | 3/361 (0.83%) | 7/359 (1.95%) | 0/354 (0.00%) | 1/349 (0.29%) | 0/394 (0.00%) | 1/259 (0.39%) | 1/426 (0.23%) | 0/276 (0.00%) |
| Sepsis | Infections and infestations | 4/361 (1.11%) | 5/359 (1.39%) | 5/354 (1.41%) | 8/349 (2.29%) | 1/394 (0.25%) | 0/259 (0.00%) | 3/426 (0.70%) | 0/276 (0.00%) |
| Upper respiratory tract infection | Infections and infestations | 4/361 (1.11%) | 3/359 (0.84%) | 0/354 (0.00%) | 3/349 (0.86%) | 2/394 (0.51%) | 0/259 (0.00%) | 1/426 (0.23%) | 0/276 (0.00%) |
| **Metabolism and nutrition disorders (1 events)** | | | | | | | | | |
| Hypercalcaemia | Metabolism and nutrition disorders | 4/361 (1.11%) | 2/359 (0.56%) | 2/354 (0.56%) | 5/349 (1.43%) | 1/394 (0.25%) | 0/259 (0.00%) | 1/426 (0.23%) | 1/276 (0.36%) |
| **Musculoskeletal and connective tissue disorders (4 events)** | | | | | | | | | |
| Back pain | Musculoskeletal and connective tissue disorders | 2/361 (0.55%) | 8/359 (2.23%) | 4/354 (1.13%) | 5/349 (1.43%) | 3/394 (0.76%) | 1/259 (0.39%) | 0/426 (0.00%) | 2/276 (0.72%) |
| Musculoskeletal chest pain | Musculoskeletal and connective tissue disorders | 1/361 (0.28%) | 1/359 (0.28%) | 2/354 (0.56%) | 3/349 (0.86%) | 1/394 (0.25%) | 0/259 (0.00%) | 1/426 (0.23%) | 0/276 (0.00%) |
| Pain in extremity | Musculoskeletal and connective tissue disorders | 1/361 (0.28%) | 0/359 (0.00%) | 0/354 (0.00%) | 2/349 (0.57%) | 1/394 (0.25%) | 0/259 (0.00%) | 1/426 (0.23%) | 0/276 (0.00%) |
| Pathological fracture | Musculoskeletal and connective tissue disorders | 5/361 (1.39%) | 4/359 (1.11%) | 17/354 (4.80%) | 25/349 (7.16%) | 4/394 (1.02%) | 1/259 (0.39%) | 3/426 (0.70%) | 2/276 (0.72%) |
| **Neoplasms benign, malignant and unspecified (incl cysts and polyps) (4 events)** | | | | | | | | | |
| Adenocarcinoma of colon | Neoplasms benign, malignant and unspecified (incl cysts and polyps) | 3/361 (0.83%) | 1/359 (0.28%) | 2/354 (0.56%) | 0/349 (0.00%) | 0/394 (0.00%) | 1/259 (0.39%) | 1/426 (0.23%) | 1/276 (0.36%) |
| Basal cell carcinoma | Neoplasms benign, malignant and unspecified (incl cysts and polyps) | 2/361 (0.55%) | 3/359 (0.84%) | 14/354 (3.95%) | 7/349 (2.01%) | 2/394 (0.51%) | 0/259 (0.00%) | 3/426 (0.70%) | 4/276 (1.45%) |
| Plasma cell myeloma | Neoplasms benign, malignant and unspecified (incl cysts and polyps) | 3/361 (0.83%) | 3/359 (0.84%) | 3/354 (0.85%) | 2/349 (0.57%) | 2/394 (0.51%) | 2/259 (0.77%) | 5/426 (1.17%) | 2/276 (0.72%) |
| Prostate cancer | Neoplasms benign, malignant and unspecified (incl cysts and polyps) | 1/361 (0.28%) | 1/359 (0.28%) | 4/354 (1.13%) | 1/349 (0.29%) | 1/394 (0.25%) | 1/259 (0.39%) | 0/426 (0.00%) | 1/276 (0.36%) |
| **Nervous system disorders (1 events)** | | | | | | | | | |
| Transient ischaemic attack | Nervous system disorders | 1/361 (0.28%) | 0/359 (0.00%) | 2/354 (0.56%) | 2/349 (0.57%) | 0/394 (0.00%) | 1/259 (0.39%) | 2/426 (0.47%) | 1/276 (0.36%) |
| **Renal and urinary disorders (1 events)** | | | | | | | | | |
| Urinary retention | Renal and urinary disorders | 0/361 (0.00%) | 2/359 (0.56%) | 1/354 (0.28%) | 0/349 (0.00%) | 0/394 (0.00%) | 1/259 (0.39%) | 1/426 (0.23%) | 0/276 (0.00%) |
| **Respiratory, thoracic and mediastinal disorders (3 events)** | | | | | | | | | |
| Chronic obstructive pulmonary disease | Respiratory, thoracic and mediastinal disorders | 2/361 (0.55%) | 1/359 (0.28%) | 3/354 (0.85%) | 0/349 (0.00%) | 2/394 (0.51%) | 0/259 (0.00%) | 2/426 (0.47%) | 0/276 (0.00%) |
| Dyspnoea | Respiratory, thoracic and mediastinal disorders | 3/361 (0.83%) | 4/359 (1.11%) | 5/354 (1.41%) | 0/349 (0.00%) | 1/394 (0.25%) | 0/259 (0.00%) | 0/426 (0.00%) | 2/276 (0.72%) |
| Pleural effusion | Respiratory, thoracic and mediastinal disorders | 2/361 (0.55%) | 0/359 (0.00%) | 0/354 (0.00%) | 1/349 (0.29%) | 2/394 (0.51%) | 0/259 (0.00%) | 1/426 (0.23%) | 0/276 (0.00%) |

**Supplementary Table 4: ‘Other’ (non-serious) Adverse Events that are Consistently Reported Across All 4 TOURMALINE Clinical Trials. Source:** clinicaltrials.gov

| **Adverse Event** | **Category** | **MM1 Ixa** | **MM1 Placebo** | **MM2 Ixa** | **MM2 Placebo** | **MM3 Ixa** | **MM3 Placebo** | **MM4 Ixa** | **MM4 Placebo** |
| --- | --- | --- | --- | --- | --- | --- | --- | --- | --- |
| **Blood and lymphatic system disorders (1 events)** | | | | | | | | | |
| Anaemia | Blood and lymphatic system disorders | 123/361 (34.07%) | 105/359 (29.25%) | 109/354 (30.79%) | 107/349 (30.66%) | 28/394 (7.11%) | 8/259 (3.09%) | 31/426 (7.28%) | 19/276 (6.88%) |
| **Gastrointestinal disorders (5 events)** | | | | | | | | | |
| Constipation | Gastrointestinal disorders | 125/361 (34.63%) | 99/359 (27.58%) | 151/354 (42.66%) | 143/349 (40.97%) | 41/394 (10.41%) | 21/259 (8.11%) | 36/426 (8.45%) | 21/276 (7.61%) |
| Diarrhea | Gastrointestinal disorders | 186/361 (51.52%) | 153/359 (42.62%) | 215/354 (60.73%) | 163/349 (46.70%) | 135/394 (34.26%) | 61/259 (23.55%) | 102/426 (23.94%) | 35/276 (12.68%) |
| Dyspepsia | Gastrointestinal disorders | 33/361 (9.14%) | 31/359 (8.64%) | 26/354 (7.34%) | 38/349 (10.89%) | 13/394 (3.30%) | 15/259 (5.79%) | 24/426 (5.63%) | 7/276 (2.54%) |
| Nausea | Gastrointestinal disorders | 114/361 (31.58%) | 83/359 (23.12%) | 133/354 (37.57%) | 98/349 (28.08%) | 154/394 (39.09%) | 40/259 (15.44%) | 119/426 (27.93%) | 23/276 (8.33%) |
| Vomiting | Gastrointestinal disorders | 93/361 (25.76%) | 47/359 (13.09%) | 107/354 (30.23%) | 46/349 (13.18%) | 105/394 (26.65%) | 28/259 (10.81%) | 102/426 (23.94%) | 11/276 (3.99%) |
| **General disorders (4 events)** | | | | | | | | | |
| Asthenia | General disorders | 62/361 (17.17%) | 66/359 (18.38%) | 94/354 (26.55%) | 97/349 (27.79%) | 30/394 (7.61%) | 17/259 (6.56%) | 25/426 (5.87%) | 17/276 (6.16%) |
| Fatigue | General disorders | 113/361 (31.30%) | 103/359 (28.69%) | 110/354 (31.07%) | 105/349 (30.09%) | 79/394 (20.05%) | 43/259 (16.60%) | 49/426 (11.50%) | 28/276 (10.14%) |
| Oedema peripheral | General disorders | 97/361 (26.87%) | 76/359 (21.17%) | 172/354 (48.59%) | 116/349 (33.24%) | 34/394 (8.63%) | 11/259 (4.25%) | 37/426 (8.69%) | 16/276 (5.80%) |
| Pyrexia | General disorders | 55/361 (15.24%) | 71/359 (19.78%) | 63/354 (17.80%) | 49/349 (14.04%) | 78/394 (19.80%) | 37/259 (14.29%) | 44/426 (10.33%) | 13/276 (4.71%) |
| **Infections and infestations (2 events)** | | | | | | | | | |
| Nasopharyngitis | Infections and infestations | 90/361 (24.93%) | 86/359 (23.96%) | 75/354 (21.19%) | 84/349 (24.07%) | 91/394 (23.10%) | 69/259 (26.64%) | 36/426 (8.45%) | 18/276 (6.52%) |
| Upper respiratory tract infection | Infections and infestations | 97/361 (26.87%) | 83/359 (23.12%) | 58/354 (16.38%) | 64/349 (18.34%) | 100/394 (25.38%) | 54/259 (20.85%) | 69/426 (16.20%) | 32/276 (11.59%) |
| **Metabolism and nutrition disorders (1 events)** | | | | | | | | | |
| Decreased appetite | Metabolism and nutrition disorders | 51/361 (14.13%) | 42/359 (11.70%) | 79/354 (22.32%) | 79/349 (22.64%) | 21/394 (5.33%) | 17/259 (6.56%) | 36/426 (8.45%) | 13/276 (4.71%) |
| **Musculoskeletal and connective tissue disorders (4 events)** | | | | | | | | | |
| Arthralgia | Musculoskeletal and connective tissue disorders | 79/361 (21.88%) | 71/359 (19.78%) | 99/354 (27.97%) | 104/349 (29.80%) | 105/394 (26.65%) | 43/259 (16.60%) | 61/426 (14.32%) | 30/276 (10.87%) |
| Back pain | Musculoskeletal and connective tissue disorders | 97/361 (26.87%) | 82/359 (22.84%) | 90/354 (25.42%) | 101/349 (28.94%) | 77/394 (19.54%) | 47/259 (18.15%) | 62/426 (14.55%) | 33/276 (11.96%) |
| Myalgia | Musculoskeletal and connective tissue disorders | 22/361 (6.09%) | 16/359 (4.46%) | 38/354 (10.73%) | 33/349 (9.46%) | 22/394 (5.58%) | 14/259 (5.41%) | 22/426 (5.16%) | 8/276 (2.90%) |
| Pain in extremity | Musculoskeletal and connective tissue disorders | 54/361 (14.96%) | 41/359 (11.42%) | 68/354 (19.21%) | 79/349 (22.64%) | 32/394 (8.12%) | 26/259 (10.04%) | 25/426 (5.87%) | 13/276 (4.71%) |
| **Nervous system disorders (3 events)** | | | | | | | | | |
| Dizziness | Nervous system disorders | 58/361 (16.07%) | 43/359 (11.98%) | 58/354 (16.38%) | 67/349 (19.20%) | 33/394 (8.38%) | 18/259 (6.95%) | 27/426 (6.34%) | 16/276 (5.80%) |
| Headache | Nervous system disorders | 53/361 (14.68%) | 56/359 (15.60%) | 48/354 (13.56%) | 58/349 (16.62%) | 43/394 (10.91%) | 23/259 (8.88%) | 23/426 (5.40%) | 11/276 (3.99%) |
| Peripheral sensory neuropathy | Nervous system disorders | 88/361 (24.38%) | 61/359 (16.99%) | 106/354 (29.94%) | 84/349 (24.07%) | 41/394 (10.41%) | 23/259 (8.88%) | 66/426 (15.49%) | 25/276 (9.06%) |
| **Psychiatric disorders (1 events)** | | | | | | | | | |
| Insomnia | Psychiatric disorders | 82/361 (22.71%) | 106/359 (29.53%) | 100/354 (28.25%) | 95/349 (27.22%) | 31/394 (7.87%) | 12/259 (4.63%) | 26/426 (6.10%) | 12/276 (4.35%) |
| **Respiratory, thoracic and mediastinal disorders (1 events)** | | | | | | | | | |
| Cough | Respiratory, thoracic and mediastinal disorders | 73/361 (20.22%) | 65/359 (18.11%) | 81/354 (22.88%) | 61/349 (17.48%) | 87/394 (22.08%) | 55/259 (21.24%) | 31/426 (7.28%) | 19/276 (6.88%) |
| **Skin and subcutaneous tissue disorders (2 events)** | | | | | | | | | |
| Pruritus | Skin and subcutaneous tissue disorders | 45/361 (12.47%) | 32/359 (8.91%) | 53/354 (14.97%) | 42/349 (12.03%) | 35/394 (8.88%) | 19/259 (7.34%) | 24/426 (5.63%) | 12/276 (4.35%) |
| Rash maculo-papular | Skin and subcutaneous tissue disorders | 34/361 (9.42%) | 15/359 (4.18%) | 80/354 (22.60%) | 40/349 (11.46%) | 24/394 (6.09%) | 9/259 (3.47%) | 36/426 (8.45%) | 2/276 (0.72%) |
| **Vascular disorders (1 events)** | | | | | | | | | |
| Hypertension | Vascular disorders | 29/361 (8.03%) | 27/359 (7.52%) | 35/354 (9.89%) | 27/349 (7.74%) | 21/394 (5.33%) | 19/259 (7.34%) | 26/426 (6.10%) | 16/276 (5.80%) |

**Supplementary Table 5: Overall Safety Data Summary. Source: clinicaltrials.gov**

| **Safety Measure** | **TOURMALINE-MM1**  **(R/R MM + LenDex)** | | **TOURMALINE-MM2**  **(NTE + LenDex ≤18 cycles + ≥19 cycles)** | | **TOURMALINE-MM3**  **(Post-ASCT Maint)** | | **TOURMALINE-MM4**  **(NDMM Maint)** | |
| --- | --- | --- | --- | --- | --- | --- | --- | --- |
|  | **Ixazomib**  **(n=361)** | **Placebo**  **(n=359)** | **Ixazomib**  **(n=354)** | **Placebo**  **(n=349)** | **Ixazomib**  **(n=395)** | **Placebo**  **(n=261)** | **Ixazomib**  **(n=425)** | **Placebo**  **(n=281)** |
| **All-cause mortality** | 250 (69.3%) | 251 (69.9%) | 158 (44.6%) | 170 (48.7%) | 144 (36.5%) | 93 (35.6%) | 184 (43.3%) | 115 (40.9%) |
|  | **Ixazomib**  **(n=361)** | **Placebo**  **(n=359)** | **Ixazomib**  **(n=354)** | **Placebo**  **(n=349)** | **Ixazomib**  **(n=394)** | **Placebo**  **(n=259)** | **Ixazomib**  **(n=426)** | **Placebo**  **(n=276)** |
| **Any TEAE** | 359 (99.4%) | 357 (99.4%) | 354 (100%) | 349 (100%) | 382 (97.0%) | 241 (93.1%) | 82 (19.2%) | 92 (33.3%) |
| **Any SAE** | 205 (56.8%) | 201 (56.0%) | 244 (68.9%) | 224 (64.2%) | 108 (27.4%) | 51 (19.7%) | 17 (3.99%) | 24 (8.70%) |

NTE = Non-transplant eligible patients

ASCT = Autologous Stem Cell Transplant

R/R = Relapsed/Refractory

MM = Multiple myeloma

All-cause mortality= ITT Population included all participants who were randomized and had post-randomization data.

Serious and Other Adverse Events: Safety population included all participants who received at least 1 dose of ixazomib or placebo. Safety populations reflect actual drug receipt and may differ slightly from ITT populations. All percentages calculated based on safety population denominators shown in headers.

**Supplementary Table 6: Ixazomib Revenue from 2016-2024**

| Fiscal Year | Ixazomib Revenue (USD Million) | Medicare Expenditure (USD million) |
| --- | --- | --- |
| 2016 | 51.56 |  |
| 2017 | 353.05 |  |
| 2018 | 547.77 |  |
| 2019 | 723.07 | 398.05 |
| 2020 | 921.73 | 466.39 |
| 2021 | 893.63 | 444.88 |
| 2022 | 789.22 | 384.90 |
| 2023 | 664.43 | 331.27 |
| 2024 | 608.67 |  |
| Total | 5553.13 | 2,025.49 |
